# Progress of oncolytic virotherapy for neuroblastoma

**DOI:** 10.3389/fped.2022.1055729

**Published:** 2022-11-18

**Authors:** Xiao-Tong Chen, Shu-Yang Dai, Yong Zhan, Ran Yang, De-Qian Chen, Yi Li, En-Qing Zhou, Rui Dong

**Affiliations:** Department of Pediatric Surgery, Children's Hospital of Fudan University, and Shanghai Key Laboratory of Birth Defects, Shanghai, China

**Keywords:** oncolytic virus, neuroblastoma, mechanism, preclinical, clinical, combination therapy

## Abstract

As a neuroendocrine tumor derived from the neural crest, neuroblastoma (NB) is the most common extracranial solid tumor in children. The prognosis in patients with low- and intermediate-risk NB is favorable while that in high-risk patients is often detrimental. However, the management of the considerably large proportion of high-risk patients remains challenging in clinical practice. Among various new approaches, oncolytic virus (OV) therapy offers great advantages in tumor treatment, especially for high-risk NB. Genetic modified OVs can target NB specifically without affecting normal tissue and avoid the widespread drug resistance issue in anticancer monotherapy. Meanwhile, its safety profile provides great potential in combination therapy with chemo-, radio-, and immunotherapy. The therapeutic efficacy of OV for NB is impressive from bench to bedside. The effectiveness and safety of OVs have been demonstrated and reported in studies on children with NB. Furthermore, clinical trials on some OVs (Celyvir, Pexa-Vec (JX-594) and Seneca Valley Virus (NTX-010)) have reported great results. This review summarizes the latest evidence in the therapeutic application of OVs in NB, including those generated in cell lines, animal models and clinical trials.

## Introduction

As a neuroendocrine tumor originating from the neural crest, neuroblastoma (NB) can occur anywhere along with the sympathetic nervous system, with the adrenal gland being the most common primary site (1). As the most common extracranial solid tumor in children, NB is the most commonly diagnosed (about 36%) oncological disease in patients under one year old. It's usually diagnosed at a very young age with a median age of 17 months, and nearly 75%–90% of cases are found in children younger than 5 years old (2, 3). Interestingly, NB often regresses spontaneously, and it's regarded as one of the tumors with the highest rate of spontaneous and complete regression (3–5). With recent development in treatment modalities such as surgical resection, chemotherapy, radiation therapy, stem cell transplantation, targeted therapy and immunotherapy, the 5-year survival of NB has been significantly improved, especially among children diagnosed at the age of 1 to 4 years (1, 6). However, these treatment modalities come with side effects and increasing likelihood of drug resistance. Although cure rates among low-risk patients are acceptable, recurrence and metastasis occur in about 60%–70% of high-risk NB patients, which are often refractory to current therapies and associated with dismal prognosis (7). Improving the prognosis in high-risk NB remains a major unmet clinical need currently. However, new insights into the role of oncolytic virus (OV) treatment may provide new hope.

Using OVs as therapeutic strategies for malignant tumors, including NB, has drawn substantial attention from various studies. OVs can be divided into two categories: natural oncolytic viruses and genetically modified ones. The commonly used wildtype oncolytic viruses are reovirus, Newcastle disease virus, parvovirus, etc, which recognize tumor cells based on their highly expressed tumor-specific receptors or abnormal intracellular signaling pathways and metabolic status, such as a defect in interferon (IFN) signaling pathway or activated Ras pathway (8–10). Most available are genetically modified OVs in which tumor tropism is attenuated to reduce virulence for non-neoplastic host cells (11, 12). Genetically modified OVs can be obtained through many ways, most commonly by deleting virulence genes to improve safety and inserting foreign genes to improve tumor targeting ability or antitumor efficacy (9). Inserting targeting peptides or specific envelope glycoproteins can endow common virus tumor tropism, such as the protein transduction domain (PTD) of the HIV-1 Tat protein (Tat-PTD) (13). Besides, targeting abnormal pathways or products in tumor cells, such as inserting tumor-specific promoters or microRNA-targeting sequences, can also be a helpful way to improve tumor targeting capabilities of viruses (9, 14). Some tumors may share similar genetic alterations, for example, rearrangement of the human telomerase reverse transcriptase (hTERT) is one of the common characteristics of many tumors including NB. This indicates that driving viruses by hTERT promoter can help tumor targeting (15, 16). Apart from hTERT rearrangement, other genetic lesions of NB such as MYCN amplification, ALK mutation, chromosomal loss (1p, 3p, 11q) or gain (17q), and p53 inactivation can also be promising targets in designing OVs (17). Genetically-modified OV bears the advantages of good targeting capability, high killing rate, less adverse reactions and lower cost. Since OVs are less dependent on specific receptor expression patterns so as to avoid the resultant mutational or transcriptional resistance that may accompany, they are less prone to develop drug resistant issues compared with conventional oncological therapies (11). Due to the high safety and multi-pathway oncolytic mechanisms, OVs have shown great potential in the field of combination therapy. When used in combination with chemotherapy and radiotherapy, OVs may assert synergistic effect and significantly enhance the anti-tumor effect, especially when combined with immunotherapy such as immune checkpoint inhibitors like anti-CTLA-4 (cytotoxic T lymphocyte-associated antigen-4) antibody and anti-PD-L1/PD-1 antibody (18). OVs infection stimulates anti-cancer immune responses that augment the efficacy of checkpoint inhibition (19).

**Figure 1:**
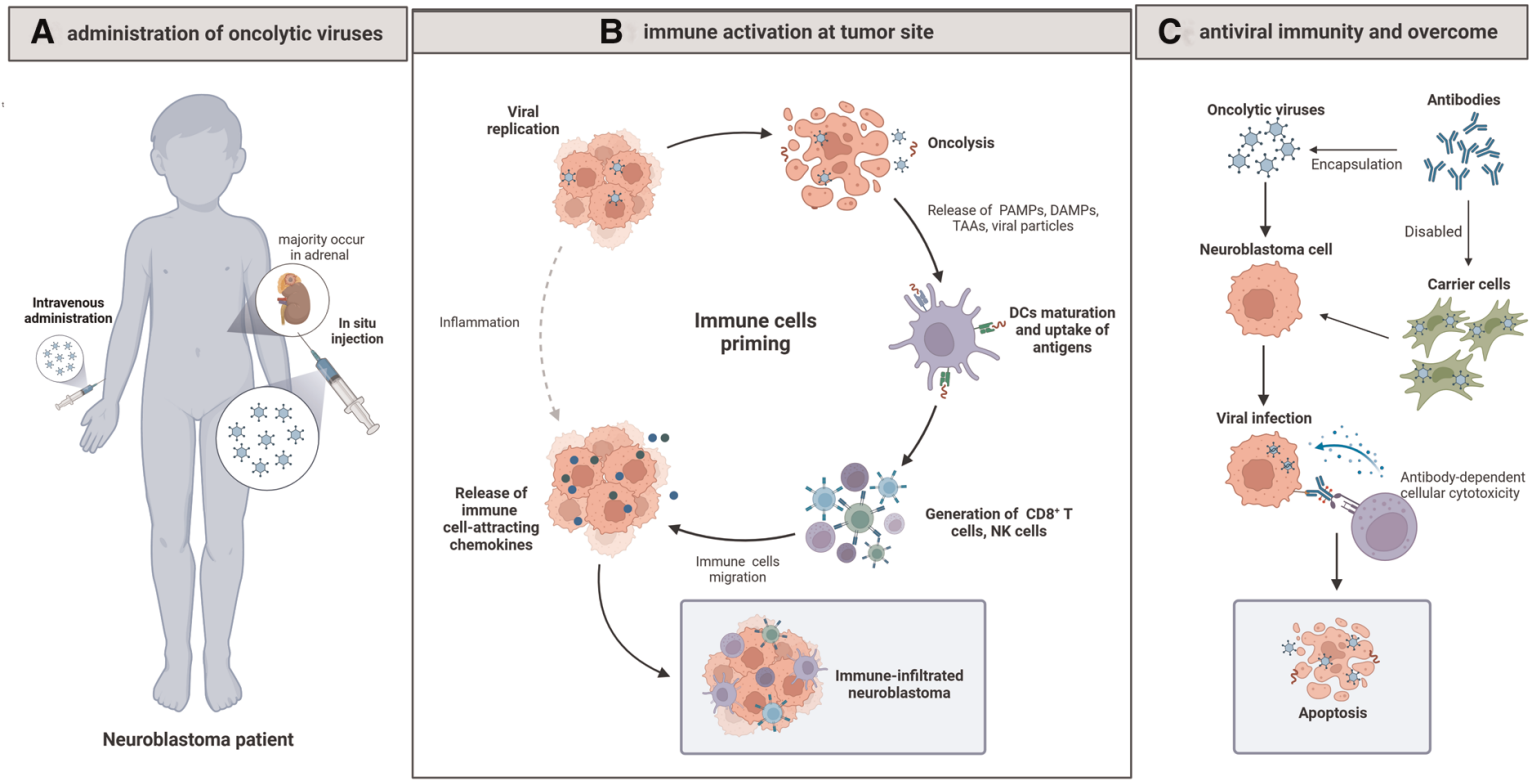
Oncolytic virotherapy for neuroblastoma. (**A**) The *in situ* injected or intravenously administrated OVs reach the NB site and cause oncolysis by cytopathic effect, causing cell lysis, cell apoptosis and necrosis. (**B**) The released PAMPs, DAMPs and TAAs activate CD8 + T cells after presentation by dendritic cells, and the released immune cell-attracting chemokines recruit NK cells, CD8 + cells and dendritic cells, changing the suppressive TME into an active one and contributing to the immune-infiltrated neuroblastoma. (**C**) Delivering OVs with carrier cells can overcome antiviral immunity effectively and improve the efficacy of OVs.

Currently, progress in OV research is expected to bring new insight into novel therapy against oncologic diseases including NB. Studies on OV therapy for NB have developed rapidly in number and depth. Some oncolytic viruses have shown good therapeutic effects in clinical trials currently, however, OV therapy in general remains to be premature and many challenges remain to be overcome. We hereby review the current status of oncolytic virotherapies for the treatment of NB (Figure 1), with focuses on preclinical studies (Table 1) and clinical trials (Table 2) on different OVs for NB.

**Table 1:** Preclinical studies of oncolytic virotherapy for neuroblastoma.

Virus	Modifications	In vitro studies: NB cell lines	In vivo studies: animal model	Results	References
Adenovirus	ZD55-shMYCN	Adenovirus ZD55 carrying short hairpin RNA (shRNA) targeting MYCN gene	LA1-55N, IMR-32, LA1-55n/Dox-R	LA1-55N s.c. xenografts in nude mice	Inhibited proliferation by downregulating MYCN expression, induced apoptosis by upregulating RKIP, inhibited xenograft growth in nude mice and re-sensitized to doxorubicin by downregulating MRP expression.	(51), (52), (53)
	OBP-301	Expression of the E1A and E1B genes linked with an internal ribosome entry site under hTERT promoter.	IMR-32, CHP-134, NB-1, LA-N-5, SK-N-SH	CHP-134 s.c. xenografts in BALB/c-nu/nu mice	Suppressed MYCN expression by activation of E2F1 protein, cytopathic effect *in vitro* by inducing autophagy, suppressed the growth of xenografts *in vivo*.	(16)
	OBP-702	Inserting a human wild-type p53 gene expression cassette driven by the Egr-1 promoter into the E3 region of OBP-301.				
	Ad-endoNF	Ablating fiber knob, integrating endosialidaseNF (endoNF) into adenoviral capsids.	IMR-32	IMR-32 s.c. xenografts in nude mice	Lysed IMR-32 cells *in vitro* and inhibited the growth of tumor xenografts in mice.	(56)
	Celyvir	Autologous marrow-derived MSCs carrying ICOVIR-5, a new Ad△24RGD-derivative oncolytic adenovirus controlled by E2F promoter which can selectively replicate in cancer cells *via* activating Rb/E2F pathway.	N/A	C57BL/6 murine adenocarcinoma models	Celyvir induced systemic immune response and intratumoral leukocyte infiltration in mice after intraperitoneal injection.	(58)
HSV-1	G207	*γ*_1_34.5 loci deletion (both copies), Escherichia coli lacZ gene insertion in the ICP6 gene.	N18 cell line	N18 s.c. xenografts in A/J mice	Killed N18 cells, inhibited subcutaneous N18 tumor growth in A/J mice.	(70)
	NV1066	γ_1_34.5 loci deletion (one copy), eGFP expression under a promoter from cytomegalovirus.	IMR-32, SKNBE (2), LAN-5, SHSY5Y, SMS-SAN, SKNSH, CHLA-20, CHLA-79	CHLA-20 and LAN-5 s.c. xenografts in nude mice	Induced apoptosis in NB cells, inhibited tumor growth *in vivo* and prolonged survival.	(72)
	HCMV/HSV-1 chimeric virus (C130 and C134)	γ_1_34.5 loci deletion (both copies), HCMV TRS1 (C130) or IRS1 (C134) expression under an HCMV immediate early promoter.	Neuro-2a	Neuro-2a s.c. xenografts in A/J mice	Improved survival in syngeneic murine Neuro-2a model.	(73)
	HSV1716	γ_1_34.5 loci deletion (both copies).	CHLA-20, CHLA-90, CHLA-119, CHLA-136, CHP-134, IMR-32, SK-N-SH, NB-1643, NB-EBc1, SK-N-AS, SK-N-BE (2), SH-SY5Y	CHLA-20, CHP-134, SK-N-AS and SK-N-BE (2) s.c. xenografts in nude mice	Sensitivity of different NB cells varied widely both *in vitro* and *in vivo*, which was not necessarily correlated to the expression of virus entry receptors but the cell-autonomous anti-viral responses.	(78)
	M002 and vvD54-M002	γ_1_34.5 loci deletion (both copies), IL-12 expression.	Neuro-2a, SK-N-SH, SK-N-AS, SK-N-BE (2), SH-SY5Y, SHEP (MYCN-), WAC2 (MYCN+)	Neuro-2a xenografts in the right cerebral hemisphere in A/J mice	Expressed IL-12, decreased survival of NB cells, inhibited tumor growth *in vivo* and prolonged survival.	(34), (35), (82), (83)
	M012	γ_1_34.5 loci deletion (both copies), bacterial cytosine deaminase expression under Egr-1 promoter.	Neuro-2a	Neuro-2a s.c. xenografts in A/J mice	Expressed cytosine deaminase, converted 5-FC to 5-FU, inhibited tumor *in vitro* and *in vivo*.	(84)
	rQT3	γ_1_34.5 loci deletion (both copies), Timp3 gene insertion into the UL39 locus.	LA-N-5, IMR-32, SKNBE (2), CHP-134, SHSY5Y, SKNSH, CHLA-20, CHLA-79	LA-N-5 s.c. xenografts in nude mice	Expressed TIMP3, reduced MMP activity, inhibited vasculogenesis, delayed tumor growth.	(85)
HSV-2	FusOn-H2	Protein kinase domain deletion of the ICP10 gene, green fluorescent protein gene insertion.	Neuro-2A	Neuro-2a s.c. xenografts in A/J mice	Induced tumor-specific CTL responses, inhibited tumor growth in virto and *in vivo*.	(88), (89)
Poliovirus	A133Gmono-crePV	A point mutation (A103G) introduction into a “spacer region” between the cloverleaf and IRES in the 5’-NTR, a cre element introduction into the 5'-nontranslated genomic region.	Neuro-2a^CD155^	Neuro-2a^CD155^ s.c. xenografts in CD155 tgA/J mice.	No signs of paralysis, elimination of tumors in CD155 tgA/J mice, construction of antitumor immunity.	(93), (94)
Measles virus	MV-CEA	CEA expression in MVEdm, an attenuated Edmonston vaccine strain.	SK-N-SH, SMS-KCNR, primary NB cells	SK-N-SH s.c. xenografts in nude mice	Expressed soluble human CEA, induced cell lysis and apoptosis, induced regression of xenografts.	(104)
Parvovirus	H-1PV	wild-type	BE (2)-C, IMR-32, Kelly, SH-EP, SH-SY5Y, SK-N-AS, SK-N-DZ, SK-N-FI, SK-N-MC, SK-N-SH, WAC-2	N/A	H-1PV was nontoxic for normal cells, induced lytic and apoptosis in NB cells.	(106)
Vesicular stomatitis virus	VSV23	Insertion of IL-23 gene.	NB41A3	N/A	Expressed IL-23, induced cytopathic effect in NB41A3 cells.	(107)
	VSV*Δ*M51	Deletion of methionine 51 in the M protein, insertion of an extra cistron encoding green fluorescent protein between the G and L sequences.	IMR-5, IMR-32, LAN-1, SK-N-SH, SK-N-AS, SH-EP, TET-21N	N/A	N-myc amplification augmented susceptibility to VSV*Δ*M51 by downregulating ISGs.	(109)
Vaccinia virus	vvDD	Deletions of the thymidine kinase and vaccinia growth factor genes.	SKNAS, SKNBE2, IMR5, IMR32	SKNAS s.c. xenografts in nude mice	SKNAS and IMR5 cells are sensitive while IMR32 cells appear to be resistant. Inhibited growth of tumors *in situ* and metastatic in mice.	(114)
	OVV-CXCR4-A-Fc	Deletions of the thymidine kinase and vaccinia growth factor genes. Insertion of lac-Z gene, eGFP and the CXCR4-A-Fc.	NXS2	NXS2 s.c. xenografts in A/J mice	Induced apoptosis of NXS2 cells. Inhibited growth of NXS2 tumors.	(115)
	VV-GD2m-NAP	Insertions of GD2m gene and NAP gene.	NXS2	NXS2 s.c. xenografts in A/J mice	Inhibited tumor growth and prolonged survival.	(116)
Sindbis virus	AR339	wild type	SK-NSH, IMR-32, LAN-5, GOTO, RT-BM-1	SK-N-SH and IMR-32 s.c. xenografts in A/J mice	Induced cytopathic effect in NB cells. Induced regression tumors *in vivo*.	(117)
Zika virus	PRVABC59	A Puerto Rican strain isolated from a human in 2015.	IMR32, SMS-KAN, SK-N-Be (1), SK-N-AS, LA-N-6, CHLA-42	N/A	Induced cytopathic effects in NB cells.	(121)

s.c., subcutaneous; N/A, not applicable; MRP, muti-drug resistance-associated protein; eGFP, enhanced green fluorescent protein; HCMV, human cytomegalovirus; ISGs, IFN-stimulated genes.

**Table 2:** Clinical trials/ case reports of oncolytic virotherapy for neuroblastoma.

Virus	Modifications	Clinical trials/ case reports	Results	References
Adenovirus	Ad5/3- Cox2LD24	A 24 bp deletion in Rb binding site of E1A. E1A gene expression under Cox2 promoter.	Case report: A six-year-old boy with high risk NB metastatic to lymph nodes and bone marrow.	The primary tumor had regressed by 71% after one month of treatment. The patient remained alive in good health after 14 months of treatment.	(126)
	Celyvir	Autologous marrow-derived MSCs carrying ICOVIR-5, a new Ad△24RGD-derivative oncolytic adenovirus controlled by E2F promoter which can selectively replicate in cancer cells *via* activating Rb/E2F pathway.	Case reports: four children with stage IV neuroblastoma; 12 children with refractory neuroblastoma. Phase I (NCT01844661): 19 adult and 15 pediatric patients (4 diagnosed with NB).	Two patients with NB showed disease stabilization after the treatment. The result of this trial suggests that Celyvir is safe and warrants further evaluation in a phase 2 setting.	(128), (129), (130), (127)
Vaccinia virus	Pexa-Vec (JX-594)	Thymidine kinase gene deletion and GM-CSF gene and lac-Z gene insertion.	Phase I (NCT01169584): 2 NB patients aged 4 and 6 respectively, who ever received high-dose chemotherapy with autologous stem cell rescue.	All toxicities were less than or equal grade 3. Tumor of the patient aged 4 was stable while that of the patient aged 20 was progressing.	(110)
Seneca Valley Virus	NTX-010 (SVV-001)	wild type	Phase I (NCT01048892): 9 NB patients of different ages and severity.	Induced cytopathic effect in NB cells. Induced regression of xenografts. Feasible and well tolerated and no DLT IN patients.	(124), (131)

Cox2: cyclo-oxygenase 2.

## The mechanism of OV's oncolytic action

OVs exert anti-tumor effect by inducing selective tumor cell cytopathological effect and establishing antitumor immunity. Besides, some engineered OVs can express therapeutic genes to significantly augment their efficacy. It has been proved that oncolytic virus can disrupt tumor angiogenesis and thereby cutting nutritional and oxygen supply to tumor cells and inhibiting tumor growth (20, 21).

### Lysis of tumor cells

OVs can lyse tumor cells directly through oncolysis and the released progeny viruses would continue to infect tumor cells, thereby achieving the cascade amplification effect of oncolysis. The highly expressed specific receptors on tumor cells are the molecular mechanism for OVs to target and lysis tumor cells, while the high metabolic state of tumor cells would facilitate viral replication (8). As we will describe elaborately in section 2.3, in addition to the natural ability of OVs in tumor cell lysis, further modifications can increase their lytic ability and target capability.

### Changes of tumor microenvironment

Another important mechanism of OVs' action is to trigger antitumor immunity and subsequently change tumor microenvironment (TME). Interestingly, OVs can induce two types of overlapping but distinct immunity: anti-tumoral and anti-viral immunities (22). These two seems contradictory in the action of OVs, and there have been debates about whether the immune system is a friend or foe to OVs. Tumor cells may have evolved to evade immune-mediated recognition and destruction while acquire the defects *via* cellular anti-viral pathways such as those mediated by the interferons (IFNs) and thereby become more susceptible to OVs (22, 23). The lysed tumor cells release viral particles, pathogen-associated molecular patterns, tumor associated or specific antigens, tumor cell debris and danger signals, which may activate the corresponding lymphocytes after being presented by dendritic cells, leading to changes of the type, number and activation status of tumor-infiltrating lymphocytes and subsequently drive innate and adaptive anti-cancer immune responses (18, 23). Besides, type I IFN secreted by lymphocytes in the classical anti-viral response can also stimulate immune cells within the TME, such as NK cells and CD8+ T cells, thus promoting the anti-tumor immune reactions (22, 24). The immune-suppressive TME is transformed into an immune-stimulating state, allowing effector T cells to enter the tumor bed and kill tumor cells (25). Meanwhile, NK cells and other immune components also play important roles in enhancing the therapeutic efficacy of OVs (26–30). In addition, the “antibody-mediated complement-dependent cancer cell lysis” is also an important mechanism of OV therapy (31). Nevertheless, the pre-existing neutralizing anti-viral antibodies, as well as the innate and adaptive anti-viral immunity, will reduce the viral load at the tumor site and the antitumor efficacy would thus be impaired (26). As a double-edged sword, immune components such as NK cells and INF may kill OVs while activating TME. As we will discuss later, this is still a hurdle for oncolytic therapy, and the real challenge is to achieve the ideal balance to maximize the therapeutic effect.

### Expression of therapeutic genes

Furthermore, the modified OVs could express cytokines to boost tumor immunity, pro-apoptotic proteins or toxin proteins coded by suicide genes to directly kill tumor cells, extracellular matrix (ECM)-degrading enzymes to increase the intra-tumoral spread of OVs, and factors to inhibit tumor angiogenesis (9, 32). Arming OVs with cytokine genes is one of the most commonly used approaches. The expression of cytokines like granulocyte macrophage-colony stimulating factor (GM-CSF), IL-2, IL-12, and tumor necrosis factor, etc. could recruit corresponding lymphocytes and thereby enhancing immune responses (32, 33). As a type of engineered HSV expressing IL-12, M002 is demonstrated to replicate in NB cells conditionally. The expressed IL-12 would induce a helper T cell subset type 1 response, thus inducing more durable antitumor effects (34, 35). Besides, OVs can also be designed to encode tumor-associated antigens expressing T cell co-stimulatory molecules or T cell checkpoint molecules that block T cell suppression (32). The combination with immune checkpoint inhibitors may induce tumor inflammation and lead to lymphocytic infiltration and migration to distant sites, and exert antitumor effect in distant (non-virally injected) tumors without distant virus spread (36). Relevant studies have shown that CD8 + and CD4 + cells can infiltrate distal lesions independent of NK cells and IFN I, and even influence distal tumor cells with similar molecular structure, thereby achieving systemic anti-tumor immunity. Although OVs armed with therapeutic genes are not widely available at present, they could be promising in future treatment of NB.

## Preclinical evidence on OVs in NB treatment

Preclinical evidence is the basis in the development of a novel treatment modality. NB cell line is an economical and practical model for preclinical research, which can be divided into different groups according to genetic lesions such as MYCN copy number amplification, ALK activation mutations, chromosome loss or increase, and TERT rearrangement (17, 37). Besides, organoids which can recapitulate the intrinsic heterogeneity of primary NB as *in vitro* models are useful to test OVs for personalized treatments (38).

Because cell lines and organoids cannot provide systematic information of interaction between body cells and OVs, establishing preclinical animal models is the key step to link laboratory finding with clinical trials (39). The commonly used humanized mouse models are cell-derived xenograft (CDX) and patient-derived tumor xenograft (PDX) models. Compared with the traditional CDX model, PDX model can reflect the original features of patient tumors and genetic diversity accurately, which is a great advantage and would vastly improve both basic and clinical study outcomes (40, 41). Furthermore, the TME of neuroblastoma PDXs contains many components that are critical for cancer progression and metastasis, including tumor-associated macrophages, fibroblasts, pericytes, endothelial cells and extracellular matrix components (40). However, the humanized mouse models also have inevitable limitations: abnormal immune system of the host mice could lead to ignorance of the body's antiviral immune response and the role of immune system in assisting OVs. Therefore, taking immunological factors into consideration, the genetically engineered mouse models (GEMMs) can also be an alternative approach. Weiss et al. developed transgenic mouse in which MYCN were overexpressed in neuroectodermal cells and neuroblastoma was induced under the control of tyrosine hydroxylase (Th) promoter (42). The Th-MYCN transgenic mouse model is generally considered as the most widely used and the best characterized neuroblastoma GEMMs. Besides, neuroblastoma GEMMs of ALK-mutant (ALKR1275 (43%) and ALKF1174 (30%)) and LIN28B overexpression (or LIN28B amplification) have also been reported (43). A major advantage of GEMMs is the adoption of immune-competent host that allows assessment of immunotherapies that stimulate tumor-specific immune responses. But owing to its genetic uniformity, GEMM cannot fully represent the diversity of neuroblastoma.

Different neuroblastoma modeling approaches have their strengths and weaknesses in various applications. Currently cell lines and CDX models remain to be the most widely used models in the study of oncolytic therapies for neuroblastoma. Hereby we introduce the following modified OVs *via* various mechanisms based on the genetic characteristics of NB, which are extremely promising in the treatment of NB based on preclinical data (Table 1).

### Adenovirus

Adenovirus (Ad) is a double-stranded DNA virus without envelope and the most commonly used viral vector for gene delivery at present (44) due to its fully knowable virus genetic elements and the possibility of manipulating them (45). At least 70 serotypes of human adenovirus exist, of which 57 have been found in humans and serotype 5 is the most commonly used due to its favorable safety and stability properties, as well as accessibility of the genome for genetic modifications (45, 46). Ad cell entry is mediated by binding with the chimeric antigen receptor (CAR) on the cell surface. Cell tropism of human adenoviruses differs among serotypes, but CAR is usually ubiquitously expressed in healthily differentiated tissue instead of tumor cells (47–49). Therefore, genetic modification by editing E1A, E1B or E3 gene might be necessary to endow adenovirus with tumor tropism.

As a member of the proto-oncogene myc family, MYCN plays a significant role in differentiation, apoptosis, cell proliferation, angiogenesis and metastasis of NB. MYCN amplification is found in 18%–38% of cases, which could increase the risk of cell migration and invasion and empower the tumor with an aggressive clinical behavior and poor survival prognosis (50). ZD55-shMYCN is an oncolytic adenovirus ZD55 carrying short hairpin RNA (shRNA) targeting the MYCN gene, which could inhibit the proliferation of LA1–55N, a p53-null and MYCN-amplified neuroblastoma cell line and suppress tumor growth in the CDX model (51). Furthermore, studies have shown that ZD55-shMYCN can induce apoptosis by upregulating RKIP in NB (52) and down-regulate the muti-drug resistance-associated protein (MRP) expression by down-regulating MYCN, thus being effective in doxorubicin-resistant NB (53). Besides MYCN amplification, human telomerase reverse transcriptase (hTERT) rearrangement can contribute to hTERT activation and poor clinical outcome, exerting a potential prognostic role in high-risk NB tumors (54, 55). OBP-301 and OBP-702 are two kinds of tumor-specific replicating adenoviruses driven by the hTERT promoter exhibiting strong therapeutic potential in four human MYCN amplified NB cell lines (IMR-32, CHP-134, NB-1, LA-N-5) and subcutaneous CHP-134 xenograft tumor model (16). The study also demonstrated that hTERT-driven oncolytic adenoviruses can downregulate MYCN expression and contribute to virus-mediated activation of E2F1 protein (16).

In addition to NB-specific genes, another major direction of Ad modification is to modify CAR-related ligands. As ligand of coxsackie and adenovirus receptor (CAR), adenoviral fiber knob is a major determinant of Ad5 for targeting. However, it's challenging to replace adenoviral fiber knobs by ligands that enable tumor specific targeting of oncolytic adenovirus because the fiber knob contributes to virus assembly (56). In a proof-of-concept study, researchers targeted high malignant tumors of neuroendocrine origin by replacing fiber knob by endosialidase NF (endoNF), i.e., the tailspike protein of bacteriophage K1F, and found that an intramolecular chaperone contained in endoNF warranted folding and compensates for the knob function in virus assembly (56). In a therapeutic mouse model of subcutaneous NB, the obtained recombinant viruses demonstrated a strong potential to inhibit tumor growth.

However, as we mentioned before, the anti-viral immunity may reduce the viral load at the tumor site and the antitumor efficacy would thus be impaired. Mesenchymal stem cells (MSCs) are ideal for protecting oncolytic virus from being killed by immune components in the blood, which enables the virus to reach the metastatic tumors and achieve tumor-targeting effect (57–59). Celyvir are autologous marrow-derived MSCs carrying ICOVIR-5, a new Ad*Δ*24RGD-derivative oncolytic adenovirus controlled by E2F promoter which can selectively replicate in cancer cells *via* activating Rb/E2F pathway (60). It's demonstrated that Ad infection could induces toll-like receptor 9 overexpression and activation of the NFB pathway in menstrual blood–derived MSCs, leading to a specific cytokine secretion profile. In vitro study showed that a proinflammatory environment would be generated when Ad-loaded MSCs were cocultured with allogeneic peripheral blood mononuclear cells, and it's mainly mediated by monocyte activation, leading to the activation of both T cells and NK cells (61). Moreover, it's demonstrated that Celyvir induced systemic immune response and intratumoral leukocyte infiltration in mice after intraperitoneal injection, and Celyvir-treated groups presented higher infiltration of CD45± cells in the core of the tumor in C57BL/6 murine adenocarcinoma models (58). Furthermore, as we will discuss later, Celyvir are being investigated in clinical trials and have shown a promising result in NB patients.

### Herpes simplex virus

Herpes simplex virus (HSV) is an enveloped double-stranded DNA virus, considered to be a therapeutic viral vector with good safety profile. Based on different antigenicity, HSV is divided into two serotypes (HSV-1 and HSV-2), and the DNA of the two virus types is 50% homologous. HSV-1 is one of the first viruses to be developed as recombinant oncolytic virus therapeutic vectors (62). HSV is a neurotropic virus whose efficacy has been widely proven by studies on brain tumors (63). Engineered HSV is also a research focus of oncolytic virus therapy for NB. All conditionally replicating HSVs currently in use contain one or more mutations within the viral genome, including thymidine kinase (64), ribonucleotide reductase (65), UTPase (66) or *γ*_1_34.5 (67). Specifically, deleting *γ*_1_34.5 genes which encodes the protein 34.5 (ICP34.5) can reduce neurotoxicity and ensure replication in actively dividing cells instead of growth arrested or terminally differentiated cells (68, 69). Furthermore, the primary entry protein for oncolytic HSV (oHSV), CD111 [poliovirus receptor-related protein 1 (PRR1, nectin-1)], is expressed by numerous NB cell lines and in human NB specimens, helping oHSV to target NB (35).

G207 is one of the earliest studied oHSVs in NB treatment. It's a conditionally replicating HSV vector created by deleting both copies of the *γ*_1_34.5 locus and inserting Escherichia coli lacZ gene which inactivates ribonucleotide reductase, an enzyme required for efficient viral DNA replication in nondividing cells but not in dividing cells (70). Its therapeutic efficacy has been demonstrated in the CDX model constructed using N18 neuroblastoma cells (70). Besides, G207 is sensitive to ganciclovir because of the retainment of an intact viral thymidine kinase (HSV-tk) gene and thereby ensuring the safety of G207 (71). NV1066 is an attenuated-engineered HSV mutant derived from strain F that has only one *γ*_1_34.5 loci deleted, with insertions of the gene encoding enhanced green fluorescent protein (eGFP) and the promoter from cytomegalovirus, making cells infected by NV1066 visible. By infecting eight NB cell lines and two different xenograft models (LAN-5 and CHLA-20) at multiple doses and time points, NV1066 has been demonstrated to be safe and very effective in lysing all the eight NB cell lines and inhibiting tumor growth (72). Furthermore, NB cell lines and tumor models are more sensitive to NV1066 than oncolytic wild type adenoviruses, making NV1066 capable to treat NB resistant to oncolytic adenovirus (72). The HCMV/HSV-1 chimeric virus, C130 and C134, are *γ*_1_34.5 deleted recombinants encoding a PKR-evasion gene (TRS1 or IRS1) from human cytomegalovirus (HCMV) (73). It ensures the virus' reductive neurovirulent and meanwhile avoids the limits of late viral protein synthesis and efficient replication in many tumors, which has been demonstrated to be superior to the parent strains (73). As one of the most studied oHSVs, HSV1716's anti-tumor efficiency has been demonstrated in many malignant tumors, including metastatic melanoma (74), oral squamous cell carcinoma (75), high-grade glioma (76) and neuroblastoma (77). *P*-y Wang et al. demonstrated the therapeutic effect of HSV1716 on NB, but the sensitivity of different NB cell lines varied widely, which was not necessarily correlated to the expression of virus entry receptors like nectin-1 but correlated to the cell-autonomous anti-viral responses (78). Besides, a recent study showed that small molecule Aurora A kinase inhibitor, alisertib, could enhance the therapeutic action of HSV1716 through cytotoxic synergy and innate cellular immune modulation, which has been demonstrated in two xenograft models of MPNST and neuroblastoma (77).

Expressing foreign genes could enhance the antitumor effect of engineered HSV-1, such as the case of cytokine-expressing viruses R8306 (IL4) (79) and R8308 (IL-10) (80) and M002 (IL-12) (34). M002 is a type of *γ*_1_34.5-negative oHSV carrying two copies of the murine interleukin-12 (IL-12) gene which expresses IL-12, a cytokine with potent antitumor properties (34, 81). It has been demonstrated that M002 infected, replicated, and decreased survival in neuroblastoma cell lines and significantly decreased tumor growth in corresponding CDX models (35, 82). Compared with the non-cytokine-expressing parent virus R3659, survival of immunocompetent A/J mice implanted with the syngeneic Neuro-2a clone of C1300 neuroblastoma tumor cells was significantly longer under the treatment of M002. Meanwhile, immunohistochemical analysis of brain tissue identified significantly more immune-related inflammatory infiltration by CD4^+^ T cells, macrophages, and CD8^+^ T cells in M002-treated tumors than that in R3659-treated tumors (34, 82). Further *in vivo* studies selected vvD54-M002 after serial passage of M002 in D54-MG tumors, which improves survival in two independent murine brain tumor models (human D54-MG intracranial xenografts in SCID mice and murine Neuro-2a neuroblastoma syngeneic tumors in A/J mice) compared to the parent (unpassaged) M002 cells (83). Besides, *in vitro* and *in vivo* studies might reveal different results, which suggests that *in vivo* tumor environment is important in selecting novel oncolytic HSV strains (83).

In addition to cytokines, expression of other therapeutic genes can also increase the oncolytic effect of oHSV. M012 is also a *γ*_1_34.5-negative oHSV with R3659 as parent mutant, which can express bacterial cytosine deaminase under the control of the cellular promoter Egr-1 (84). The expressed cytosine deaminase converts non-toxic 5-fluorocytosine (5-FC) to highly toxic, chemotherapeutic agent 5-fluorouracil (5-FU), enhancing the cytotoxicity of adjacent uninfected cells. Compared to R3659 combined with 5-FC or M012 alone, intratumoral injection of M012 in combination with 5-FC significantly reduced tumor growth (84). rQT3 is an engineered *γ*_1_34.5-negative oHSV with human Timp3 gene inserted into the UL39 locus, thus expressing human tissue inhibitor of metalloproteinases 3 (TIMP3), a factor that inhibits the proteolytic activity of all known matrix metalloproteinases (MMPs) within tissues (85). In many malignancies including NB, decreased expression of TIMPs often cause increased MMP expression, which could promote tumorigenesis by direct effects upon tumor cells and indirect effects upon the tumor microenvironment (86, 87). The study on rQT3 in neuroblastoma cell lines and xenograft model of NB (LA-N-5) shows that rQT3 enhanced antitumor efficacy through multiple mechanisms, including direct cytotoxicity, elevated virus titer, and reduced tumor neovascularization (85).

Although HSV-1 is much more frequently used, HSV-2 also plays a vital role in OV therapy against NB. FusOn-H2 is a mutant HSV-2 constructed by replacing the serine/threonine protein kinase domain of ICP10 with the gene encoding the green fluorescent protein, thus enabling it to selectively replicate in and lyse tumor cells under an activated Ras signaling pathway (88). The anti-tumor effects accomplished by FusOn-H2 induced syncytia formation, tumor-specific CTL responses and antitumor immunity have been demonstrated in neuro-2A murine NB cell lines and the corresponding CDX models (89).

### Poliovirus

Poliovirus, the pathogen of paralytic poliomyelitis, is a nonenveloped positive-stranded RNA virus which rarely invades the central nervous system but targets predominantly motor neurons, causing flaccid paralysis and even death. It's considered a promising novel virus in oncolytic therapies (90). But the potential neurovirulence and the high coverage of vaccine against polio in early childhood are challenging. Therefore, reducing the neurovirulence of poliovirus is a prerequisite for its application. Hidemi Toyoda et al. tested the effects of live-attenuated poliovirus vaccine, Sabin 1, 2 and 3 strains (Japan Poliomyelitis Research Institute), in 29 established NB cell lines and an animal model established using athymic mice transplanted with SJ-N-JF NB cells (91). A total of 27 of 29 established neuroblastoma cell lines were killed and tumors on both the inoculated and contralateral flank regressed completely and dramatically (91). Thus, the curative effect of Poliovirus on NB was preliminarily demonstrated.

A133Gmono-crePV is a novel and stable poliovirus attenuated by introducing a point mutation (A103G) in a “spacer region” between the cloverleaf and IRES in the 5′-NTR (92). The attenuating mutation A103G in the spacer region was unstable on replication. Nevertheless, the introduced cre element, a stem-loop structure mapping to the coding region of viral protein2C^ATPase^, interrupts the spacer region, making A133Gmono-crePV a stable attenuation phenotype that replicates in NB cells (93). The mouse NB cells (Neuro-2aCD155) expressing CD155, the poliovirus receptor, are subcutaneously implanted into the CD155 tgA/J mice which is immunized against poliovirus to build a relatively perfect model. Despite the pre-existing high titers of anti-poliovirus antibodies, it has been demonstrated that A133Gmono-crePV eliminated the Neuro2aCD155 tumors in CD155 tgA/J mice (93). Furthermore, the *in vivo* destruction of neuroblastoma cells by A133Gmono-crePV can lead to a robust antitumor immune response mediated by cytotoxic CD8+ T cells, because no tumor growth was observed after reinoculation of Neuro-2aCD155 in the cured mice and the adoptive transfer of splenocytes obtained from cured mice markedly delayed the growth of previously established NB tumors (94).

### Measles virus

Measles virus (MV) is an enveloped virion that contains a non-segmented, negative-stranded RNA genome, belonging to the Mononegavirales order and Paramyxoviridae family. MV enters cells through the interaction of the H-glycoprotein with the MV receptors, including CD150 (signaling lymphocyte-activation molecule, SLAM), CD46 and nectin-4 (95). Furthermore, MV has neuronal tropism and can infect cells *via* the possible SLAM negative and CD46-independent pathway, causing various types of neurological disease (96). MVEdm is attenuated Edmonston vaccine strain of the measles virus which has been proven to be safe and effective as oncolytic therapy for many tumors (97–99). Compared to wildtype MV that enters cells mainly through the SLAM receptor, MVEdm enters cells through CD46 receptor, which is frequently overexpressed on tumors (100, 101). Besides, the nectin-4 receptor may also be helpful for the entrance of MVEdm (102). The characteristics mentioned above make MV, especially MVEdm, potentially useful in treating NB.

Shucheng Zhang et al. investigated the capability of the recombinant MV-Edm that expresses the carcinoembryonic antigen (MV-CEA) against NB. MV-CEA is a kind of trackable oncolytic measles viruses expressing inert (nonimmunogenic, nonfunctional and accurately measurable) soluble human carcinoembryonic antigen (hCEA), which didn't compromise virus replication (103). Expression of the recombinant genes in viruses was easily monitored by measuring the concentrations of hCEA in tissue culture supernatant or in the serum. Studies found that despite the low expression of CD46 and nectin-4, MV-CEA can successfully replicate in the human NB cell lines and xenografts. Intratumoral administration of MV-CEA can induce significant apoptosis in NB xenografts and prolong the survival of tumor-laiden animals (104).

### Parvovirus

Some rodent parvoviruses are found with natural oncolytic and onco-suppressive activities, and H-1PV is one of them. H-1PV is a nonenveloped rat parvovirus with viral capsid containing a linear single-stranded DNA molecule of about 5 kb. It has been proven to be safe and tolerable *in vitro* and *in vivo* in the treatment of a variety of tumors such as glioma, pancreatic carcinoma, breast cancer and colorectal cancer (105). As the first naturally occurring wild-type virus applied to NB that doesn't need genetical modification to acquire tumor tropism, H-1PV was demonstrated to be non-toxic for nontransformed cells and selectively lyse NB cells in 11 NB cell lines with different MYCN status (106). Moreover, the lytic effect of H-1PV was observed to be associated with G2-arrest and induction of apoptosis, independent of MYCN oncogene amplification or differentiation status (106). The anti-neuroblastoma effect of H-1PV was promising, but still need *in vivo* evaluation in appropriate animal models and further clinical trials.

### Vesicular stomatitis virus

Vesicular stomatitis virus (VSV) is an enveloped negative-stranded RNA virus with therapeutic potential in a variety of tumors, including those with aberrant p53, Ras or Myc function such as glioblastomas, breast cancer, and NB (107). The tumors that are unable to activate immune responses including the interferon (IFN) response pathways make VSV able to specifically replicate in tumor cells. The artificial VSV23 expressing the pro-inflammatory cytokine interleukin-23 (IL-23) is significantly attenuated to avoid the possible fatal encephalitis (108). VSV23 has been demonstrated to maintain oncolytic capacity *in vitro* in multiple cell lines including NB41A3 (107). Furthermore, studies have shown that N-myc amplification in NB cell lines could augment the susceptibility to the mutant VSV*Δ*M51 by downregulating IFN-stimulated genes (109).

### Vaccinia virus

Vaccinia virus (VV) is an enveloped, double-stranded DNA virus with unique advantages as an oncolytic therapy for NB. It has been tested to be safe in different kinds of adult tumors and has undergone phase 1 and 2 trials. It has also been proven to be safe for pediatric tumors experimentally and clinically (110). Besides, effective antivirals are available to prevent the possible toxicity. Most importantly, VV was demonstrated amenable for intravenous delivery to distant tumors, making it an outstanding choice in treating metastatic tumors (111). Photodynamic therapy (PDT) could augment the efficacy of oncolytic VV against primary and metastatic tumors in mice (112). The double-deleted VV (vvDD) was engineered from Western Reserve (WR) strain by deleting the thymidine kinase and vaccinia growth factor genes to further enhance the safety profile (113). vvDD showed successful antitumor effects in several pediatric solid tumor cell lines including NB cell lines, and intravenous administration of a single dose of vvDD significantly inhibited the growth of tumors as well as metastatic tumors in xenograft models (114). The phase 1 study of Pexa-Vec (JX-594), a vaccinia virus derived from the commonly used Wyeth vaccine strain by deleting a thymidine kinase gene and inserting GM-CSF gene and lac-Z gene, showed that intratumoral injection of Pexa-Vec in pediatric patients with NB was relatively safe, but may cause transient flu-like symptoms and pustules (110).

The oncolytic VV could also be an excellent vector intravenously, such as the designed oncolytic VV(OVV-CXCR4-A-Fc) which could deliver a CXCR4 antagonist expressed in the context of the murine Fc fragment of IgG2a (115). CXCR4 was expressed in NB cell lines and the binding of CXCR4 with its ligand C-X-C motif chemokine 12 (CXCL12) may induce NB tumor cell metastasis and augment the immunosuppressive network (115). It has been demonstrated that intravenous injection of OVV-CXCR4-A-Fc could inhibit the growth of subcutaneous NXS2 NB more efficiently than oncolysis alone, which is associated with decreases in the immunosuppressive networks in the tumor microenvironment (115). A recent study showed that the constructed VV-GD2m-NAP with insertion of disialoganglioside mimotope (GD2m) and neutrophil-activating protein (NAP) genes into Western Reserve (WR) strain could significantly control subcutaneous NB (NXS2) tumor growth and prolong the survival of NXS2-tumor-bearing mice (116). Interestingly, VV-GD2m, which only expressed GD2m (a mimotope of neuroblastoma-associated glycolipid antigen), did not improve the anti-tumor capacity while VV-GD2m-NAP, which co-expresses NAP (a chemoattractant of immune cells and antigenic activator of weak immunogens), can significantly improve its therapeutic efficacy (116).

### Sindbis virus

Sindbis virus (SINV) is an RNA virus belonging to the alphavirus genus of the togaviridae family. Compared to normal cells, neoplastic cells often overexpress the 67 kDa, high-affinity laminin receptor (LR) which could enhance SINV infection as an attachment factor, promoting tumor-selective binding of SINV (117, 118). The SINV AR339 strain is relatively non-virulent and can induce the remarkable cytopathic effect in NB cell lines (SK-NSH, IMR-32, LAN-5, GOTO, and RT-BM-1) (117). The *in vivo* antitumor potential of SINV AR339 strain was also significant as intratumoral and intravenous SINV inoculations can lead to regression of NB xenograft tumors (117). As a blood-borne virus, SINV AR339 strain also has the advantage in treating metastatic tumors, making it hopeful as a novel therapy for NB.

### Zika virus

Zika virus is a neurotropic, mosquito-borne single-stranded RNA virus belonging to the flavivirus genus and the flaviviridae family (119). Zika virus infections could cause congenital microcephaly and other fetal brain defects in infants born to infected mothers (120). Nevertheless, in children and non-pregnant adults, eighty percent of Zika virus infections are asymptomatic (120). The minimal pathogenic effects of natural Zika virus infection in children ensure its safety as oncolytic therapy on NB (121). Joseph Mazar et al. confirmed that Zika virus strain PRVABC59 could infect both MYCN-amplified [IMR32, SMS-KAN, and SK-N-Be(1)] and non-MYCN-amplified NB cells (SK-N-AS, LA-N-6, and CHLA-42), which was directly correlated with the expression of the cell surface glycoprotein CD24 (121). The result suggests its potential targeting therapeutic role in NB treatment.

### Seneca valley virus

Seneca Valley virus (SVV) is an oncolytic RNA virus belonging to the Senecavirus genus of the Picornaviridae family, which can selectively infect and lyse tumor cells with neuroendocrine features. As a relatively small virion (25–30 nm), SVV allows greater distribution to tumor which is not inhibited by human blood components and systemic delivery to treat metastatic diseases (122). Tumor cell lines possessing neuroendocrine properties, including multidrug-resistant NB cell lines which express neuroendocrine markers, were sensitive to Seneca Valley Virus-001 (SVV-001) (123), also known as Seneca Valley virus (NTX-010) (124). The *in vitro* testing result showed that 3 of 4 neuroblastoma cell lines (NB-1643, NB-EBd, CHLA-136, but not CHLA-90) were sensitive to NTX-010, and anti-tumor effect in the NB xenografts models kicked in after intravenous injection of viral particles (124).

## Clinical trials of OVs on neuroblastoma

Clinical trials on several OVs, including vaccinia virus, adenovirus, reovirus, herpes simplex virus, and measles virus have been conducted in adults for different kinds of tumors. Combining with chemotherapy, radiotherapy and immunotherapy, especially immune checkpoint inhibitors, OV is an effective therapy in improving the therapeutic effect of cancer treatments (125). However, clinical trials in pediatric tumors remains insufficient, with only a few sporadic cases using OVs to treat children with NB being reported.

The earliest case reports of oncolytic adenovirus used in children with refractory NB were published in 2010. It reported a six-year-old boy with high risk non-4S stage 4 disease metastasized to the lymph nodes and bone marrow who has undergone a series of failed treatments including three different chemotherapy regimens and a high-dose intensive regimen with an autologous stem cell transplant (126). The patient was treated with an ultrasound-guided injection of 10e11 viral particles of Ad5/3-Cox2LD24, an engineered oncolytic adenovirus which acquired tumor tropism by the cyclo-oxygenase 2 (Cox2) promoter driving E1A gene and a 24-bp deletion in Rb binding site of E1A, into the primary tumor site near the left kidney, adjacent lymph nodes and intravenously. One month after the treatment, the primary tumor had regressed by 71% while after three months of treatment, the bone marrow biopsy showed a minimal number of tumor cells. Ad5/3-Cox2LD24 effectively replicated and disseminated within the tumor despite the rapid induction of neutralizing antibodies. Moreover, CD3+/CD8 + cytotoxic lymphocytes increased after the treatment, which indicated the induction of antitumor immunity (126). Importantly, after 14 months of treatment by Ad5/3-Cox2LD24, the patient remained alive in good health status (Table 2).

As a promising systemically delivered OV, Celyvir showed great potential in clinical studies because the use of MSCs could increase the amount of ICOVIR-5 administered to patients, minimize toxicities and avoid direct tumor injections (127). The earliest case of Celyvir use in NB was reported in four children with stage IV neuroblastoma refractory to front-line therapies, in which all patients received at least two doses of Celyvir infusions (128). The tolerance to the treatment was excellent. The peripheral presence of ICOVIR-5 was verified in all patients, and it was detected in marrow aspirate in one patient 5 days after the second Celyvir infusion, suggesting that Celyvir carried ICOVIR-5 to areas with metastasis. Importantly, thirty-six months after the first Celyvir treatment and more than 4 years after diagnosis, this patient was in complete remission. Due to the significant therapeutic effect of Celyvir in the first case report, researchers compassionately used Celyvir in treating 12 children diagnosed with advanced metastatic neuroblastoma (129). The tolerance was excellent, with very mild and self-limited viral-related symptoms. After receiving weekly multidose of Celyvir without any concomitant treatment, the clinical outcomes varied among these patients (8 had disease progression, 1 remained stable, 3 had partial response and 1 had complete response), and it's associated with the difference of patients' MSCs. The biopsies on the patient who received the maximum doses of Celyvir infusions showed that the primary tumor site had more infiltrated immune cells and stronger immunogenicity 4 months after initiating Celyvir therapy (130). Recently, the Celyvir researchers presented the results of the first-in-human, first-in child trial of Celyvir (NCT01844661) (127). They recruited 15 pediatric and 19 adult patients initially but only 9 pediatric (4 diagnosed with NB) and 7 adult patients received treatment with Celyvir eventually mainly because rapid disease progression before Celyvir was available. Adenoviral replication detected by PCR was found in all but 2 pediatric patient and in none of the adult ones. Two patients with NB showed disease stabilization after the treatment. The result of this trial suggests that Celyvir is safe and warrants further evaluation in a phase 2 setting.

Another OV that have gone through clinical trials in treating NB is Pexa-Vec (JX-594), an engineered vaccinia virus described above (110). In a phase I clinical trial (NCT01169584) 6 patients were enrolled, of which 2 were diagnosed with NB and had previously received high-dose chemotherapy with autologous stem cell rescue. All the recruited patients were greater than 1 year of age and less than 22 years of age with recurrent or refractory non–central nervous system solid tumors with no known curative pathway. The tolerance was excellent, with all toxicities ≤ grade 3. Twenty-two days after intratumoral injection of Pexa-Vec, the tumor of one 4-year-old NB patient was stable while that of another 20-year-old NB patient had progressed. However, differed from the adult Pexa-Vec trials in which antitumor immunity was measured by detecting complement-dependent antitumor antibodies in serum, this pediatric trial did not show any changes in noninjected lesions that might have resulted from an immunologic effect possibly. This might be due to the relatively short time frame that the patients were observed in this study prior to seeking other treatments. Overall, this study suggests that Pexa-Vec is safe in pediatric patients via intratumoral injection and further studies are warranted (Table 2).

Furthermore, the Seneca Valley Virus NTX-010 has also undergone phase I trial (NCT01048892) in children (131). Patients (≥3 to ≤21 years) with neuroblastoma, rhabdomyosarcoma, or rare tumors with neuroendocrine features were eligible. Thirteen patients (9 with NB) enrolled in one arm received treatment of s single-dose NTX-010 while 9 patients (3 with NB) enrolled another arm received treatment of two doses of NTX-010 in combination with cyclophosphamide (CTX) orally and intravenously. The result showed that NTX-010 was feasible and well tolerated and no dose-limiting toxicity (DLT) was observed. The main adverse effects included tumor-related pain and lymphopenia. Despite the combination with CTX, the neutralizing antibodies were still generated and viruses in both the blood circulation and the stool were cleared after NTX-010 infusion. Therefore, the use of NTX-010 by intravenous injection may play a potential role in NB treatment which needs further validations (Table 2).

## Challenges and future perspectives

Although the therapeutic role of OVs in the treatment of NB is promising, there are also multiple challenges need to be addressed. As case reports and clinical trials have shown, there are some adverse effects of OVs during NB treatment such as fever, diarrhea, stomach pain, and liver enzyme elevations, which are usually self-limited and milder than those reported in other existing therapies (57–60, 126–131). There are also risks of uncontrolled virus replication *in vivo* and possible transmission to patients' contacts (132). But in general, oncolytic virotherapy is currently safe without presented potential safety issues that cannot be eliminated (132). However, there are still many challenges to be solved. For instance, the presence of intracellular junctions and extracellular matrix may be the barriers for OVs to spread and penetrate the tumor bulk. Pre-treatment with collagenase or co-administration of hyaluronidase with oncolytic adenoviruses may facilitate the spread of OVs (20, 133, 134). But the insurmountable hurdles of OVs remain to be anti-viral immune responses, off-target infection and uncertain efficacy (20).

One of the biggest challenges is the elimination of OVs by the immune system. Compared to intraperitoneal, intracranial, intrapleural, and limb perfusion, systemic delivery is the most feasible and simplified administration route to deliver OVs to the distant sites in systemic diseases (135). However, systemic delivery is associated with anti-viral immune responses, potential off-target effects and the need for much higher doses (135). The systemically delivered OVs may be neutralized by the pre-existing or therapy-induced neutralizing antibodies and removed by the reticuloendothelial system (136). Due to the prevalent existence of antibodies against adenovirus, HSV, measles virus, poliovirus, and vaccinia virus in people, OVs are mainly injected intratumorally to achieve the effective therapeutic concentration, which sometimes limits the use of OVs to tumors close to the body surface. Thus, the treatment of metastatic diseases with OVs is challenging (125). However, the most common primary site of NB is the adrenal gland, which is deep inside the body cavity. In addition, NB is one of the tumors with the highest recurrence and metastasis rates, making it necessary to deliver OVs systemically. Several studies have shown that the prevalence of HSV-1 seropositivity rises with age, and that under 20% in children aged 1–4 in the United States have HSV premunition (63). This suggests that younger children may be better candidates for systemic therapies. As described above, there are many new types of OVs discovered that can be delivered systemically. One clinical trial of oncolytic poxvirus JX-594 showed that it could infect, replicate, and expresse transgene products in the cancer tissue in a dose-related fashion after intravenous infusion (111). Sindbis virus (117) and Seneca Valley virus (123) mentioned above are also promising in treating metastatic tumors due to the low prevalence of pre-existing antibodies in the human blood. Besides, MSCs was found to be an ideal delivery vehicle to protect OVs from being attenuated by components in the blood, as well as recruited T cells (137). Consistently, clinical studies on Celyvir, an MSC-delivered adenovirus, have reported good results, which is a significant step forward for OVs in the clinical treatment of NB.

Furthermore, due to the heterogeneity of NB and different mechanisms of action among various OV types, the response to NB also vary according to the limited clinical data (110, 127, 129, 130). Considering the high price of oncolytic drugs, it's necessary to screen whether specific OVs are suitable for specific NB patients. However, there's no robust predictive biomarkers yet to forecast patients' response to oncolytic virotherapy (20). But the developing of NB organoid is a promising model for personalized oncolytic viruses testing *in vitro* due to its efficiency and sensitivity (138).

Although challenge and hurdles remain, the development of oncolytic therapeutics is gaining momentum, especially in combination therapies with chemotherapy, radiation and immunotherapy against NB. ZD55-shMYCN (described above) was demonstrated to be able to re-sensitize the doxorubicin-resistant neuroblastoma by down-regulating MYCN and thus down-regulating the multi-drug resistance-associated protein (MRP) expression (53). Therefore, the combination of ZD55-shMYCN with doxorubicin provides a novel therapeutic strategy for treating doxorubicin-resistant NB, which is a clinically significant progress. The combination of immune checkpoint inhibitors (ICIs) with OVs for cancer therapy is a hot area. OVs can transform the “cold” tumor microenvironment with few immune effector cells into a “hot” one with increased immune cell and cytokine infiltration, which will help to increase the effectiveness of ICIs (19). Many ongoing clinical trials combining OVs with ICIs like CTLA-4, PD-1 and other ICIs have been reported, but none of them are studying their effect in NB treatment (19). Besides, synergizing with chimeric antigen receptor (CAR)-modified T cells can significantly augment the therapeutic efficacy of OV, overcome multiple challenges, and attract more CAR-T cells by altering the immune suppressive TME (139).

## Conclusion

Overall, OV has brought new hope to NB as a cancer that is difficult to cure. Various types of genetically modified OVs have shown good therapeutic effects in NB cell lines. However, further studies are still needed in NB organoids and PDX models which can better character the original NB and more accurately predict its clinical effects. At present, studies on OVs have been reported in many sporadic NB cases, and some have also entered phase I clinical trial stage with good results despite some mild side effects. The effect of oncolytic therapy for NB is generally favorable from bench to bedside, but further high-quality clinical trials with more pediatric NB subjects are still in demand to evaluate the safety and effectiveness of OVs. Besides, some severe challenges remain, and further studies should focus on identifying effective delivery methods, exploring the mechanism of OV on positive regulation of the immune system, and promoting the combination of OVs with ICIs and CAR-T therapy. It is imperative to adopt multiple strategies to overcome the myriad obstacles in translating these findings into clinic practice. With these obstacles to be solved one day, oncolytic virotherapy is expected to be an adjunctive therapy for NB that can be combined with gene therapy, immunotherapy, radiotherapy and chemotherapy.

## References

[1] Mahapatra S Challagundla KB. Neuroblastoma. StatPearls. Treasure Island (FL): StatPearls Publishing Copyright © 2020, StatPearls Publishing LLC (2020).

[2] Leis K Baska A Bereźnicka W Marjańska A Mazur E Lewandowski BT Resveratrol in the treatment of neuroblastoma: a review. Rev Neurosci. (2020) 31(8):873–81. 10.1515/revneuro-2020-0021 32920543

[3] Maris JM. Recent advances in neuroblastoma. N Engl J Med. (2010) 362(23):2202–11. 10.1056/NEJMra0804577 20558371 PMC3306838

[4] Yamamoto K Hanada R Kikuchi A Ichikawa M Aihara T Oguma E Spontaneous regression of localized neuroblastoma detected by mass screening. J Clin Oncol. (1998) 16(4):1265–9. 10.1200/jco.1998.16.4.1265 9552024

[5] Nakagawara A, Li YY, Izumi H, Muramori K, Inada H, Nishi M. Neuroblastoma. *Jpn J Clin Oncol*. (2018) 48(3):214–41. 10.1093/jjco/hyx176 29378002

[6] Linabery AM Ross JA. Childhood and adolescent cancer survival in the US by race and ethnicity for the diagnostic period 1975-1999. Cancer. (2008) 113(9):2575–96. 10.1002/cncr.23866 18837040 PMC2765225

[7] Hartmann O Valteau-Couanet D Vassal G Lapierre V Brugières L Delgado R Prognostic factors in metastatic neuroblastoma in patients over 1 year of age treated with high-dose chemotherapy and stem cell transplantation: a multivariate analysis in 218 patients treated in a single institution. Bone Marrow Transplant. (1999) 23(8):789–95. 10.1038/sj.bmt.1701737 10231141

[8] Lawler SE Speranza MC Cho CF Chiocca EA. Oncolytic viruses in cancer treatment: a review. JAMA Oncol. (2017) 3(6):841–9. 10.1001/jamaoncol.2016.2064 27441411

[9] Lan Q Xia S Wang Q Xu W Huang H Jiang S Development of oncolytic virotherapy: from genetic modification to combination therapy. Front Med. (2020) 14(2):160–84. 10.1007/s11684-020-0750-4 32146606 PMC7101593

[10] Miest TS Cattaneo R. New viruses for cancer therapy: meeting clinical needs. Nat Rev Microbiol. (2014) 12(1):23–34. 10.1038/nrmicro3140 24292552 PMC4002503

[11] Raja J Ludwig JM Gettinger SN Schalper KA Kim HS. Oncolytic virus immunotherapy: future prospects for oncology. J Immunother Cancer. (2018) 6(1):140. 10.1186/s40425-018-0458-z 30514385 PMC6280382

[12] Russell SJ Peng KW Bell JC. Oncolytic virotherapy. Nat Biotechnol. (2012) 30(7):658–70. 10.1038/nbt.2287 22781695 PMC3888062

[13] Yu D Jin C Leja J Majdalani N Nilsson B Eriksson F Adenovirus with hexon Tat-protein transduction domain modification exhibits increased therapeutic effect in experimental neuroblastoma and neuroendocrine tumors. J Virol. (2011) 85(24):13114–23. 10.1128/JVI.05759-11 21957304 PMC3233137

[14] Menotti L Cerretani A Hengel H Campadelli-Fiume G. Construction of a fully retargeted herpes simplex virus 1 recombinant capable of entering cells solely via human epidermal growth factor receptor 2. J Virol. (2008) 82(20):10153–61. 10.1128/jvi.01133-08 18684832 PMC2566291

[15] Fujiwara T Shirakawa Y Kagawa S. Telomerase-specific oncolytic virotherapy for human gastrointestinal cancer. Expert Rev Anticancer Ther. (2011) 11(4):525–32. 10.1586/era.10.200 21504319

[16] Tanimoto T Tazawa H Ieda T Nouso H Tani M Oyama T Elimination of MYCN-amplified neuroblastoma cells by telomerase-targeted oncolytic virus via MYCN suppression. Mol Ther Oncolytics. (2020) 18:14–23. 10.1016/j.omto.2020.05.015 32637577 PMC7321810

[17] Harenza JL Diamond MA Adams RN Song MM Davidson HL Hart LS Transcriptomic profiling of 39 commonly-used neuroblastoma cell lines. Sci Data. (2017) 4:170033. 10.1038/sdata.2017.33 28350380 PMC5369315

[18] Locy H de Mey S de Mey W De Ridder M Thielemans K Maenhout SK. Immunomodulation of the tumor microenvironment: turn foe into friend. Front Immunol. (2018) 9:2909. 10.3389/fimmu.2018.02909 30619273 PMC6297829

[19] Sivanandam V LaRocca CJ Chen NG Fong Y Warner SG. Oncolytic viruses and immune checkpoint inhibition: the best of both worlds. Mol Ther Oncolytics. (2019) 13:93–106. 10.1016/j.omto.2019.04.003 31080879 PMC6503136

[20] Goradel NH Baker AT Arashkia A Ebrahimi N Ghorghanlu S Negahdari B. Oncolytic virotherapy: challenges and solutions. Curr Probl Cancer. (2021) 45(1):100639. 10.1016/j.currproblcancer.2020.100639 32828575

[21] Breitbach CJ Arulanandam R De Silva N Thorne SH Patt R Daneshmand M Oncolytic vaccinia virus disrupts tumor-associated vasculature in humans. Cancer Res. (2013) 73(4):1265–75. 10.1158/0008-5472.CAN-12-2687 23393196

[22] Lemos de Matos A Franco LS McFadden G. Oncolytic viruses and the immune system: the dynamic duo. Mol Ther Methods Clin Dev. (2020) 17:349–58. 10.1016/j.omtm.2020.01.001 32071927 PMC7015832

[23] Filley AC Dey M. Immune system, friend or foe of oncolytic virotherapy? Front Oncol. (2017) 7:106. 10.3389/fonc.2017.00106 28589085 PMC5440545

[24] Achard C Surendran A Wedge ME Ungerechts G Bell J Ilkow CS. Lighting a fire in the tumor microenvironment using oncolytic immunotherapy. EBioMedicine. (2018) 31:17–24. 10.1016/j.ebiom.2018.04.020 29724655 PMC6013846

[25] Gujar S Pol JG Kim Y Lee PW Kroemer G. Antitumor benefits of antiviral immunity: an underappreciated aspect of oncolytic virotherapies. Trends Immunol. (2018) 39(3):209–21. 10.1016/j.it.2017.11.006 29275092

[26] Chaurasiya S Chen NG Fong Y. Oncolytic viruses and immunity. Curr Opin Immunol. (2018) 51:83–90. 10.1016/j.coi.2018.03.008 29550660 PMC9285655

[27] Bhat R Dempe S Dinsart C Rommelaere J. Enhancement of NK cell antitumor responses using an oncolytic parvovirus. Int J Cancer. (2011) 128(4):908–19. 10.1002/ijc.25415 20473905

[28] Ogbomo H Zemp FJ Lun X Zhang J Stack D Rahman MM Myxoma virus infection promotes NK lysis of malignant gliomas in vitro and in vivo. PloS one. (2013) 8(6):e66825. 10.1371/journal.pone.0066825 23762498 PMC3677932

[29] Melcher A Parato K Rooney CM Bell JC. Thunder and lightning: immunotherapy and oncolytic viruses collide. Mol Ther. (2011) 19(6):1008–16. 10.1038/mt.2011.65 21505424 PMC3129809

[30] Cassady KA Haworth KB Jackson J Markert JM Cripe TP. To infection and beyond: the multi-pronged anti-cancer mechanisms of oncolytic viruses. Viruses. (2016) 8(2):43. 10.3390/v8020043 26861381 PMC4776198

[31] Kim MK Breitbach CJ Moon A Heo J Lee YK Cho M Oncolytic and immunotherapeutic vaccinia induces antibody-mediated complement-dependent cancer cell lysis in humans. Sci Transl Med. (2013) 5(185):185ra63. 10.1126/scitranslmed.3005361 23677592

[32] Bommareddy PK Shettigar M Kaufman HL. Integrating oncolytic viruses in combination cancer immunotherapy. Nat Rev Immunol. (2018) 18(8):498–513. 10.1038/s41577-018-0014-6 29743717

[33] Tahtinen S Blattner C Vaha-Koskela M Saha D Siurala M Parviainen S T-cell therapy enabling adenoviruses coding for IL2 and TNFalpha induce systemic immunomodulation in mice with spontaneous melanoma. J Immunother. (2016) 39(9):343–54. 10.1097/CJI.0000000000000144 27741089

[34] Parker JN Gillespie GY Love CE Randall S Whitley RJ Markert JM. Engineered herpes simplex virus expressing IL-12 in the treatment of experimental murine brain tumors. Proc Natl Acad Sci USA. (2000) 97(5):2208–13. 10.1073/pnas.040557897 10681459 PMC15779

[35] Gillory LA Megison ML Stewart JE Mroczek-Musulman E Nabers HC Waters AM Preclinical evaluation of engineered oncolytic herpes simplex virus for the treatment of neuroblastoma. PloS one. (2013) 8(10):e77753. 10.1371/journal.pone.0077753 24130898 PMC3795073

[36] Kaufman HL Kohlhapp FJ Zloza A. Oncolytic viruses: a new class of immunotherapy drugs. Nat Rev Drug Discov. (2015) 14(9):642–62. 10.1038/nrd4663 26323545 PMC7097180

[37] Campos Cogo S Gradowski Farias da Costa do Nascimento T de Almeida Brehm Pinhatti F de França Junior N Santos Rodrigues B Cavalli LR An overview of neuroblastoma cell lineage phenotypes and in vitro models. Exp BiolMed. (2020) 245(18):1637–47. 10.1177/1535370220949237 PMC7802384 32787463

[38] Fusco P Parisatto B Rampazzo E Persano L Frasson C Di Meglio A Patient-derived organoids (PDOs) as a novel in vitro model for neuroblastoma tumours. BMC Cancer. (2019) 19(1):970. 10.1186/s12885-019-6149-4 31638925 PMC6802324

[39] Ben-Yakar A. High-content and high-throughput in vivo drug screening platforms using microfluidics. Assay Drug Dev Technol. (2019) 17(1):8–13. 10.1089/adt.2018.908 30657702

[40] Braekeveldt N Bexell D. Patient-derived xenografts as preclinical neuroblastoma models. Cell Tissue Res. (2018) 372(2):233–43. 10.1007/s00441-017-2687-8 28924803 PMC5915499

[41] Murayama T Gotoh N. Patient-derived xenograft models of breast cancer and their application. Cells. (2019) 8(6):621. 10.3390/cells8060621 31226846 PMC6628218

[42] Weiss WA Aldape K Mohapatra G Feuerstein BG Bishop JM. Targeted expression of MYCN causes neuroblastoma in transgenic mice. EMBO J. (1997) 16(11):2985–95. 10.1093/emboj/16.11.2985 9214616 PMC1169917

[43] Kamili A Atkinson C Trahair TN Fletcher JI. Mouse models of high-risk neuroblastoma. Cancer Metastasis Rev. (2020) 39(1):261–74. 10.1007/s10555-020-09855-0 31989509

[44] Ginn SL Amaya AK Alexander IE Edelstein M Abedi MR. Gene therapy clinical trials worldwide to 2017: an update. J Gene Med. (2018) 20(5):e3015. 10.1002/jgm.3015 29575374

[45] Rius-Rocabert S García-Romero N García A Ayuso-Sacido A Nistal-Villan E. Oncolytic virotherapy in glioma tumors. Int J Mol Sci. (2020) 21(20):7604. 10.3390/ijms21207604 33066689 PMC7589679

[46] Russell L Peng KW. The emerging role of oncolytic virus therapy against cancer. Chin Clin Oncol. (2018) 7(2):16. 10.21037/cco.2018.04.04 29764161 PMC6557159

[47] Anders M Vieth M Röcken C Ebert M Pross M Gretschel S Loss of the coxsackie and adenovirus receptor contributes to gastric cancer progression. Br J Cancer. (2009) 100(2):352–9. 10.1038/sj.bjc.6604876 19142187 PMC2634721

[48] Cripe TP Wang PY Marcato P Mahller YY Lee PW. Targeting cancer-initiating cells with oncolytic viruses. Mol Ther. (2009) 17(10):1677–82. 10.1038/mt.2009.193 19672244 PMC2835002

[49] Ramírez M García-Castro J Alemany R. Oncolytic virotherapy for neuroblastoma. Discov Med. (2010) 10(54):387–93. 21122470

[50] Aygun N. Biological and genetic features of neuroblastoma and their clinical importance. Curr Pediatr Rev. (2018) 14(2):73–90. 10.2174/1573396314666180129101627 29380702

[51] Li Y Zhang B Zhang H Zhu X Feng D Zhang D Oncolytic adenovirus armed with shRNA targeting MYCN gene inhibits neuroblastoma cell proliferation and in vivo xenograft tumor growth. J Cancer Res Clin Oncol. (2013) 139(6):933–41. 10.1007/s00432-013-1406-4 23443256 PMC11824448

[52] Li Y Zhang H Zhu X Feng D Zhang D Zhuo B Oncolytic adenovirus-mediated short hairpin RNA targeting MYCN gene induces apoptosis by upregulating RKIP in neuroblastoma. Tumour Biol. (2015) 36(8):6037–43. 10.1007/s13277-015-3280-y 25736927

[53] Li Y Zhuo B Yin Y Han T Li S Li Z Anti-cancer effect of oncolytic adenovirus-armed shRNA targeting MYCN gene on doxorubicin-resistant neuroblastoma cells. Biochem Biophys Res Commun. (2017) 491(1):134–9. 10.1016/j.bbrc.2017.07.062 28711493

[54] Peifer M Hertwig F Roels F Dreidax D Gartlgruber M Menon R Telomerase activation by genomic rearrangements in high-risk neuroblastoma. Nature. (2015) 526(7575):700–4. 10.1038/nature14980 26466568 PMC4881306

[55] Valentijn LJ Koster J Zwijnenburg DA Hasselt NE van Sluis P Volckmann R TERT Rearrangements are frequent in neuroblastoma and identify aggressive tumors. Nat Genet. (2015) 47(12):1411–4. 10.1038/ng.3438 26523776

[56] Martin NT Wrede C Niemann J Brooks J Schwarzer D Kühnel F Targeting polysialic acid-abundant cancers using oncolytic adenoviruses with fibers fused to active bacteriophage borne endosialidase. Biomaterials. (2018) 158:86–94. 10.1016/j.biomaterials.2017.12.008 29304405

[57] Komarova S Kawakami Y Stoff-Khalili MA Curiel DT Pereboeva L. Mesenchymal progenitor cells as cellular vehicles for delivery of oncolytic adenoviruses. Mol Cancer Ther. (2006) 5(3):755–66. 10.1158/1535-7163.Mct-05-0334 16546991

[58] Morales-Molina Á Gambera S Cejalvo T Moreno R Rodríguez-Milla M Perisé-Barrios AJ Antitumor virotherapy using syngeneic or allogeneic mesenchymal stem cell carriers induces systemic immune response and intratumoral leukocyte infiltration in mice. Cancer Immunol Immunother. (2018) 67(10):1589–602. 10.1007/s00262-018-2220-2 30066102 PMC11028294

[59] Franco-Luzón L González-Murillo Á Alcántara-Sánchez C García-García L Tabasi M Huertas AL Systemic oncolytic adenovirus delivered in mesenchymal carrier cells modulate tumor infiltrating immune cells and tumor microenvironment in mice with neuroblastoma. Oncotarget. (2020) 11(4):347–61. 10.18632/oncotarget.27401 32064039 PMC6996901

[60] Cascallo M Alonso MM Rojas JJ Perez-Gimenez A Fueyo J Alemany R. Systemic toxicity-efficacy profile of ICOVIR-5, a potent and selective oncolytic adenovirus based on the pRB pathway. Mol Ther. (2007) 15(9):1607–15. 10.1038/sj.mt.6300239 17579575

[61] Moreno R Fajardo CA Farrera-Sal M Perisé-Barrios AJ Morales-Molina A Al-Zaher AA Enhanced antitumor efficacy of oncolytic adenovirus-loaded menstrual blood-derived mesenchymal stem cells in combination with peripheral blood mononuclear cells. Mol Cancer Ther. (2019) 18(1):127–38. 10.1158/1535-7163.Mct-18-0431 30322950

[62] Buijs PR Verhagen JH van Eijck CH van den Hoogen BG. Oncolytic viruses: from bench to bedside with a focus on safety. Hum Vaccin Immunother. (2015) 11(7):1573–84. 10.1080/21645515.2015.1037058 25996182 PMC4514197

[63] Friedman GK Pressey JG Reddy AT Markert JM Gillespie GY. Herpes simplex virus oncolytic therapy for pediatric malignancies. Mol Ther. (2009) 17(7):1125–35. 10.1038/mt.2009.73 19367259 PMC2835221

[64] Martuza RL Malick A Markert JM Ruffner KL Coen DM. Experimental therapy of human glioma by means of a genetically engineered virus mutant. Science. (1991) 252(5007):854–6. 10.1126/science.1851332 1851332

[65] Boviatsis EJ Park JS Sena-Esteves M Kramm CM Chase M Efird JT Long-term survival of rats harboring brain neoplasms treated with ganciclovir and a herpes simplex virus vector that retains an intact thymidine kinase gene. Cancer Res. (1994) 54(22):5745–51. 7954393

[66] Pyles RB Thompson RL. Evidence that the herpes simplex virus type 1 uracil DNA glycosylase is required for efficient viral replication and latency in the murine nervous system. J Virol. (1994) 68(8):4963–72. 10.1128/jvi.68.8.4963-4972.1994 8035495 PMC236437

[67] Chung RY Saeki Y Chiocca EA. B-myb promoter retargeting of herpes simplex virus gamma34.5 gene-mediated virulence toward tumor and cycling cells. J Virol. (1999) 73(9):7556–64. 10.1128/jvi.73.9.7556-7564.1999 10438845 PMC104282

[68] Holman HA MacLean AR. Neurovirulent factor ICP34.5 uniquely expressed in the herpes simplex virus type 1 Delta gamma 1 34.5 mutant 1716. J Neurovirol. (2008) 14(1):28–40. 10.1080/13550280701769999 18300073

[69] McKie EA Brown SM MacLean AR Graham DI. Histopathological responses in the CNS following inoculation with a non-neurovirulent mutant (1716) of herpes simplex virus type 1 (HSV 1): relevance for gene and cancer therapy. Neuropathol Appl Neurobiol. (1998) 24(5):367–72. 10.1046/j.1365-2990.1998.00133.x 9821167

[70] Todo T Rabkin SD Sundaresan P Wu A Meehan KR Herscowitz HB Systemic antitumor immunity in experimental brain tumor therapy using a multimutated, replication-competent herpes simplex virus. Hum Gene Ther. (1999) 10(17):2741–55. 10.1089/10430349950016483 10584921

[71] Todo T Rabkin SD Martuza RL. Evaluation of ganciclovir-mediated enhancement of the antitumoral effect in oncolytic, multimutated herpes simplex virus type 1 (G207) therapy of brain tumors. Cancer Gene Ther. (2000) 7(6):939–46. 10.1038/sj.cgt.7700182 10880026

[72] Parikh NS Currier MA Mahller YY Adams LC Di Pasquale B Collins MH Oncolytic herpes simplex virus mutants are more efficacious than wild-type adenovirus type 5 for the treatment of high-risk neuroblastomas in preclinical models. Pediatr Blood Cancer. (2005) 44(5):469–78. 10.1002/pbc.20268 15570577

[73] Shah AC Parker JN Gillespie GY Lakeman FD Meleth S Markert JM Enhanced antiglioma activity of chimeric HCMV/HSV-1 oncolytic viruses. Gene Ther. (2007) 14(13):1045–54. 10.1038/sj.gt.3302942 17429445

[74] MacKie RM Stewart B Brown SM. Intralesional injection of herpes simplex virus 1716 in metastatic melanoma. Lancet. (2001) 357(9255):525–6. 10.1016/S0140-6736(00)04048-4 11229673

[75] Mace AT Ganly I Soutar DS Brown SM. Potential for efficacy of the oncolytic herpes simplex virus 1716 in patients with oral squamous cell carcinoma. Head Neck. (2008) 30(8):1045–51. 10.1002/hed.20840 18615711

[76] Harrow S Papanastassiou V Harland J Mabbs R Petty R Fraser M HSV1716 Injection into the brain adjacent to tumour following surgical resection of high-grade glioma: safety data and long-term survival. Gene Ther. (2004) 11(22):1648–58. 10.1038/sj.gt.3302289 15334111

[77] Currier MA Sprague L Rizvi TA Nartker B Chen CY Wang PY Aurora A kinase inhibition enhances oncolytic herpes virotherapy through cytotoxic synergy and innate cellular immune modulation. Oncotarget. (2017) 8(11):17412–27. 10.18632/oncotarget.14885 28147331 PMC5392259

[78] Wang PY Swain HM Kunkler AL Chen CY Hutzen BJ Arnold MA Neuroblastomas vary widely in their sensitivities to herpes simplex virotherapy unrelated to virus receptors and susceptibility. Gene Ther. (2016) 23(2):135–43. 10.1038/gt.2015.105 26583803 PMC4742391

[79] Broberg EK Salmi AA Hukkanen V. IL-4 is the key regulator in herpes simplex virus-based gene therapy of BALB/c experimental autoimmune encephalomyelitis. Neurosci Lett. (2004) 364(3):173–8. 10.1016/j.neulet.2004.04.059 15196670

[80] Broberg EK Peltoniemi J Nygårdas M Vahlberg T Röyttä M Hukkanen V. Spread and replication of and immune response to gamma134.5-negative herpes simplex virus type 1 vectors in BALB/c mice. J Virol. (2004) 78(23):13139–52. 10.1128/jvi.78.23.13139-13152.2004 15542666 PMC525003

[81] Hellums EK Markert JM Parker JN He B Perbal B Roizman B Increased efficacy of an interleukin-12-secreting herpes simplex virus in a syngeneic intracranial murine glioma model. Neuro Oncol. (2005) 7(3):213–24. 10.1215/S1152851705000074 16053696 PMC1871915

[82] Bauer DF Pereboeva L Gillespie GY Cloud GA Elzafarany O Langford C Effect of HSV-IL12 loaded tumor cell-based vaccination in a mouse model of high-grade neuroblastoma. J Immunol Res. (2016) 2016:2568125. 10.1155/2016/2568125 27610392 PMC5005549

[83] Shah AC Price KH Parker JN Samuel SL Meleth S Cassady KA Serial passage through human glioma xenografts selects for a Deltagamma134.5 herpes simplex virus type 1 mutant that exhibits decreased neurotoxicity and prolongs survival of mice with experimental brain tumors. J Virol. (2006) 80(15):7308–15. 10.1128/jvi.00725-06 16840311 PMC1563698

[84] Guffey MB Parker JN Luckett WS Jr. Gillespie GY Meleth S Whitley RJ Engineered herpes simplex virus expressing bacterial cytosine deaminase for experimental therapy of brain tumors. Cancer Gene Ther. (2007) 14(1):45–56. 10.1038/sj.cgt.7700978 16990846

[85] Mahller YY Vaikunth SS Ripberger MC Baird WH Saeki Y Cancelas JA Tissue inhibitor of metalloproteinase-3 via oncolytic herpesvirus inhibits tumor growth and vascular progenitors. Cancer Res. (2008) 68(4):1170–9. 10.1158/0008-5472.CAN-07-2734 18281493 PMC2855837

[86] Ribatti D Surico G Vacca A De Leonardis F Lastilla G Montaldo PG Angiogenesis extent and expression of matrix metalloproteinase-2 and -9 correlate with progression in human neuroblastoma. Life Sci. (2001) 68(10):1161–8. 10.1016/s0024-3205(00)01030-4 11228100

[87] Visse R Nagase H. Matrix metalloproteinases and tissue inhibitors of metalloproteinases: structure, function, and biochemistry. Circ Res. (2003) 92(8):827–39. 10.1161/01.RES.0000070112.80711.3D 12730128

[88] Fu X Tao L Cai R Prigge J Zhang X. A mutant type 2 herpes simplex virus deleted for the protein kinase domain of the ICP10 gene is a potent oncolytic virus. Mol Ther. (2006) 13(5):882–90. 10.1016/j.ymthe.2006.02.007 16569513

[89] Li H Dutuor A Tao L Fu X Zhang X. Virotherapy with a type 2 herpes simplex virus-derived oncolytic virus induces potent antitumor immunity against neuroblastoma. Clin Cancer Res. (2007) 13(1):316–22. 10.1158/1078-0432.CCR-06-1625 17200370

[90] Gromeier M Lachmann S Rosenfeld MR Gutin PH Wimmer E. Intergeneric poliovirus recombinants for the treatment of malignant glioma. Proc Natl Acad Sci U S A. (2000) 97(12):6803–8. 10.1073/pnas.97.12.6803 10841575 PMC18745

[91] Toyoda H Ido M Hayashi T Gabazza EC Suzuki K Kisenge RR Experimental treatment of human neuroblastoma using live-attenuated poliovirus. Int J Oncol. (2004) 24(1):49–58. 10.3892/ijo.24.1.49 14654940

[92] De Jesus N Franco D Paul A Wimmer E Cello J. Mutation of a single conserved nucleotide between the cloverleaf and internal ribosome entry site attenuates poliovirus neurovirulence. J Virol. (2005) 79(22):14235–43. 10.1128/jvi.79.22.14235-14243.2005 16254358 PMC1280220

[93] Toyoda H Yin J Mueller S Wimmer E Cello J. Oncolytic treatment and cure of neuroblastoma by a novel attenuated poliovirus in a novel poliovirus-susceptible animal model. Cancer Res. (2007) 67(6):2857–64. 10.1158/0008-5472.CAN-06-3713 17363609

[94] Toyoda H Wimmer E Cello J. Oncolytic poliovirus therapy and immunization with poliovirus-infected cell lysate induces potent antitumor immunity against neuroblastoma in vivo. Int J Oncol. (2011) 38(1):81–7. 10.3892/ijo_00000826 21109928

[95] Sato H Yoneda M Honda T Kai C. Morbillivirus receptors and tropism: multiple pathways for infection. Front Microbiol. (2012) 3:75. 10.3389/fmicb.2012.00075 22403577 PMC3290766

[96] Yanagi Y Takeda M Ohno S. Measles virus: cellular receptors, tropism and pathogenesis. J Gen Virol. (2006) 87(Pt 10):2767–79. 10.1099/vir.0.82221-0 16963735

[97] Meng X Nakamura T Okazaki T Inoue H Takahashi A Miyamoto S Enhanced antitumor effects of an engineered measles virus edmonston strain expressing the wild-type N, P, L genes on human renal cell carcinoma. Mol Ther. (2010) 18(3):544–51. 10.1038/mt.2009.296 20051938 PMC2839424

[98] Grote D Russell SJ Cornu TI Cattaneo R Vile R Poland GA Live attenuated measles virus induces regression of human lymphoma xenografts in immunodeficient mice. Blood. (2001) 97(12):3746–54. 10.1182/blood.v97.12.3746 11389012

[99] Phuong LK Allen C Peng KW Giannini C Greiner S TenEyck CJ Use of a vaccine strain of measles virus genetically engineered to produce carcinoembryonic antigen as a novel therapeutic agent against glioblastoma multiforme. Cancer Res. (2003) 63(10):2462–9. 12750267

[100] Yanagi Y. The cellular receptor for measles virus. Uirusu. (2001) 51(2):201–8. 10.2222/jsv.51.201 11977762

[101] Jurianz K Ziegler S Garcia-Schuler H Kraus S Bohana-Kashtan O Fishelson Z Complement resistance of tumor cells: basal and induced mechanisms. Mol Immunol. (1999) 36(13-14):929–39. 10.1016/s0161-5890(99)00115-7 10698347

[102] Noyce RS Bondre DG Ha MN Lin LT Sisson G Tsao MS Tumor cell marker PVRL4 (nectin 4) is an epithelial cell receptor for measles virus. PLoS Pathog. (2011) 7(8):e1002240. 10.1371/journal.ppat.1002240 21901103 PMC3161989

[103] Peng KW Facteau S Wegman T O'Kane D Russell SJ. Non-invasive in vivo monitoring of trackable viruses expressing soluble marker peptides. Nat Med. (2002) 8(5):527–31. 10.1038/nm0502-527 11984600

[104] Zhang SC Cai WS Zhang Y Jiang KL Zhang KR Wang WL. Engineered measles virus edmonston strain used as a novel oncolytic viral system against human neuroblastoma through a CD46 and nectin 4-independent pathway. Cancer Lett. (2012) 325(2):227–37. 10.1016/j.canlet.2012.07.008 22796607

[105] Marchini A Bonifati S Scott EM Angelova AL Rommelaere J. Oncolytic parvoviruses: from basic virology to clinical applications. Virol J. (2015) 12:6. 10.1186/s12985-014-0223-y 25630937 PMC4323056

[106] Lacroix J Leuchs B Li J Hristov G Deubzer HE Kulozik AE Parvovirus H1 selectively induces cytotoxic effects on human neuroblastoma cells. Int J Cancer. (2010) 127(5):1230–9. 10.1002/ijc.25168 20087864

[107] Miller JM Bidula SM Jensen TM Reiss CS. Vesicular stomatitis virus modified with single chain IL-23 exhibits oncolytic activity against tumor cells in vitro and in vivo. Int J Interferon Cytokine Mediat Res. (2010) 2010(2):63–72. 10.2147/ijicmr.s9528 20556219 PMC2885733

[108] Miller J Bidula SM Jensen TM Reiss CS. Cytokine-modified VSV is attenuated for neural pathology, but is both highly immunogenic and oncolytic. Int J Interferon Cytokine Mediat Res. (2009) 1:15–32. 10.2147/ijicmr.s6776 20607123 PMC2895263

[109] Corredor JC Redding N Blote K Robbins SM Senger DL Bell JC N-Myc expression enhances the oncolytic effects of vesicular stomatitis virus in human neuroblastoma cells. Mol Ther Oncolytics. (2016) 3:16005. 10.1038/mto.2016.5 27626059 PMC5008254

[110] Cripe TP Ngo MC Geller JI Louis CU Currier MA Racadio JM Phase 1 study of intratumoral Pexa-vec (JX-594), an oncolytic and immunotherapeutic vaccinia virus, in pediatric cancer patients. Mol Ther. (2015) 23(3):602–8. 10.1038/mt.2014.243 25531693 PMC4351466

[111] Breitbach CJ Burke J Jonker D Stephenson J Haas AR Chow LQ Intravenous delivery of a multi-mechanistic cancer-targeted oncolytic poxvirus in humans. Nature. (2011) 477(7362):99–102. 10.1038/nature10358 21886163

[112] Gil M Bieniasz M Seshadri M Fisher D Ciesielski MJ Chen Y Photodynamic therapy augments the efficacy of oncolytic vaccinia virus against primary and metastatic tumours in mice. Br J Cancer. (2011) 105(10):1512–21. 10.1038/bjc.2011.429 21989183 PMC3242530

[113] Park BH Hwang T Liu TC Sze DY Kim JS Kwon HC Use of a targeted oncolytic poxvirus, JX-594, in patients with refractory primary or metastatic liver cancer: a phase I trial. Lancet Oncol. (2008) 9(6):533–42. 10.1016/s1470-2045(08)70107-4 18495536

[114] Lun X Ruan Y Jayanthan A Liu DJ Singh A Trippett T Double-deleted vaccinia virus in virotherapy for refractory and metastatic pediatric solid tumors. Mol Oncol. (2013) 7(5):944–54. 10.1016/j.molonc.2013.05.004 23816608 PMC5528451

[115] Komorowski M Tisonczyk J Kolakowska A Drozdz R Kozbor D. Modulation of the tumor microenvironment by CXCR4 antagonist-armed viral oncotherapy enhances the antitumor efficacy of dendritic cell vaccines against neuroblastoma in syngeneic mice. Viruses. (2018) 10(9):445. 10.3390/v10090455 30149659 PMC6165252

[116] Ma J Jin C Čančer M Wang H Ramachandran M Yu D. Concurrent expression of HP-NAP enhances antitumor efficacy of oncolytic vaccinia virus but not for semliki forest virus. Mol Ther Oncolytics. (2021) 21:356–66. 10.1016/j.omto.2021.04.016 34141872 PMC8182386

[117] Takenouchi A Saito K Saito E Saito T Hishiki T Matsunaga T Oncolytic viral therapy for neuroblastoma cells with Sindbis virus AR339 strain. Pediatr Surg Int. (2015) 31(12):1151–9. 10.1007/s00383-015-3784-y 26298056

[118] Formisano P Ragno P Pesapane A Alfano D Alberobello AT Rea VE PED/PEA-15 interacts with the 67 kD laminin receptor and regulates cell adhesion, migration, proliferation and apoptosis. J Cell Mol Med. (2012) 16(7):1435–46. 10.1111/j.1582-4934.2011.01411.x 21895963 PMC3823213

[119] Musso D Gubler DJ. Zika virus. Clin Microbiol Rev. (2016) 29(3):487–524. 10.1128/cmr.00072-15 27029595 PMC4861986

[120] Plourde AR Bloch EM. A literature review of Zika virus. Emerg Infect Dis. (2016) 22(7):1185–92. 10.3201/eid2207.151990 27070380 PMC4918175

[121] Mazar J Li Y Rosado A Phelan P Kedarinath K Parks GD Zika virus as an oncolytic treatment of human neuroblastoma cells requires CD24. PloS One. (2018) 13(7):e0200358. 10.1371/journal.pone.0200358 30044847 PMC6059425

[122] Burke MJ. Oncolytic seneca valley virus: past perspectives and future directions. Oncolytic Virother. (2016) 5:81–9. 10.2147/ov.S96915 27660749 PMC5019429

[123] Reddy PS Burroughs KD Hales LM Ganesh S Jones BH Idamakanti N Seneca valley virus, a systemically deliverable oncolytic picornavirus, and the treatment of neuroendocrine cancers. J Natl Cancer Inst. (2007) 99(21):1623–33. 10.1093/jnci/djm198 17971529 PMC5261858

[124] Morton CL Houghton PJ Kolb EA Gorlick R Reynolds CP Kang MH Initial testing of the replication competent seneca valley virus (NTX-010) by the pediatric preclinical testing program. Pediatr Blood Cancer. (2010) 55(2):295–303. 10.1002/pbc.22535 20582972 PMC3003870

[125] Mondal M Guo J He P Zhou D. Recent advances of oncolytic virus in cancer therapy. Hum Vaccine Immunother. (2020) 16(10):2389–402. 10.1080/21645515.2020.1723363 PMC7644205 32078405

[126] Pesonen S Helin H Nokisalmi P Escutenaire S Ribacka C Sarkioja M Oncolytic adenovirus treatment of a patient with refractory neuroblastoma. Acta Oncol. (2010) 49(1):117–9. 10.3109/02841860903071369 19735000

[127] Ruano D López-Martín JA Moreno L Lassaletta Á Bautista F Andión M First-in-human, first-in-child trial of autologous MSCs carrying the oncolytic virus icovir-5 in patients with advanced tumors. Mol Ther. (2020) 28(4):1033–42. 10.1016/j.ymthe.2020.01.019 32053771 PMC7132606

[128] García-Castro J Alemany R Cascalló M Martínez-Quintanilla J Arriero Mdel M Lassaletta A Treatment of metastatic neuroblastoma with systemic oncolytic virotherapy delivered by autologous mesenchymal stem cells: an exploratory study. Cancer Gene Ther. (2010) 17(7):476–83. 10.1038/cgt.2010.4 20168350

[129] Melen GJ Franco-Luzón L Ruano D González-Murillo Á Alfranca A Casco F Influence of carrier cells on the clinical outcome of children with neuroblastoma treated with high dose of oncolytic adenovirus delivered in mesenchymal stem cells. Cancer Lett. (2016) 371(2):161–70. 10.1016/j.canlet.2015.11.036 26655276

[130] Franco-Luzón L García-Mulero S Sanz-Pamplona R Melen G Ruano D Lassaletta Á Genetic and immune changes associated with disease progression under the pressure of oncolytic therapy in a neuroblastoma outlier patient. Cancers (Basel). (2020) 12(5):1104. 10.3390/cancers12051104 PMC7281487 32354143

[131] Burke MJ Ahern C Weigel BJ Poirier JT Rudin CM Chen Y Phase I trial of seneca valley virus (NTX-010) in children with relapsed/refractory solid tumors: a report of the children's oncology group. Pediatr Blood Cancer. (2015) 62(5):743–50. 10.1002/pbc.25269 25307519 PMC4376652

[132] Li L Liu S Han D Tang B Ma J. Delivery and biosafety of oncolytic virotherapy. Front Oncol. (2020) 10:475. 10.3389/fonc.2020.00475 32373515 PMC7176816

[133] Kuriyama N Kuriyama H Julin CM Lamborn K Israel MA. Pretreatment with protease is a useful experimental strategy for enhancing adenovirus-mediated cancer gene therapy. Hum Gene Ther. (2000) 11(16):2219–30. 10.1089/104303400750035744 11084679

[134] Ganesh S Gonzalez-Edick M Gibbons D Van Roey M Jooss K. Intratumoral coadministration of hyaluronidase enzyme and oncolytic adenoviruses enhances virus potency in metastatic tumor models. Clin Cancer Res. (2008) 14(12):3933–41. 10.1158/1078-0432.CCR-07-4732 18559615

[135] Moaven O Mangieri CW Stauffer JA Anastasiadis PZ Borad MJ. Evolving role of oncolytic virotherapy: challenges and prospects in clinical practice. JCO Precis Oncol. (2021) 5:432–41. 10.1200/po.20.00395 PMC8232075 34250386

[136] Power AT Bell JC. Cell-based delivery of oncolytic viruses: a new strategic alliance for a biological strike against cancer. Mol Ther. (2007) 15(4):660–5. 10.1038/sj.mt.6300098 17264852

[137] Rincón E Cejalvo T Kanojia D Alfranca A Rodríguez-Milla M Gil Hoyos RA Mesenchymal stem cell carriers enhance antitumor efficacy of oncolytic adenoviruses in an immunocompetent mouse model. Oncotarget. (2017) 8(28):45415–31. 10.18632/oncotarget.17557 28525366 PMC5542197

[138] Kholosy W Derieppe M van den Ham F Ober K Su Y Custers L Neuroblastoma and DIPG organoid coculture system for personalized assessment of novel anticancer immunotherapies. J Pers Med. (2021) 11(9):869. 10.3390/jpm11090869 34575646 PMC8466534

[139] Guedan S Alemany R. CAR-T cells and oncolytic viruses: joining forces to overcome the solid tumor challenge. Front Immunol. (2018) 9:2460. 10.3389/fimmu.2018.02460 30405639 PMC6207052

